# Seeing is believing: the nocturnal malarial mosquito *Anopheles coluzzii* responds to visual host-cues when odour indicates a host is nearby

**DOI:** 10.1186/s13071-016-1609-z

**Published:** 2016-06-03

**Authors:** Frances Hawkes, Gabriella Gibson

**Affiliations:** Department of Agriculture, Health & Environment, Natural Resources Institute, University of Greenwich at Medway, Chatham Maritime, Kent, ME4 4TB UK

**Keywords:** *Anopheles gambiae*, *Anopheles coluzzii*, Vision, Olfaction, Behaviour, 3D tracking, Host-seeking, Mosquito sampling

## Abstract

**Background:**

The immediate aim of our study was to analyse the behaviour of the malarial mosquito *Anopheles coluzzii* (*An. gambiae* species complex) near a human host with the ultimate aim of contributing to our fundamental understanding of mosquito host-seeking behaviour and the overall aim of identifying behaviours that could be exploited to enhance sampling and control strategies.

**Results:**

Based on 3D video recordings of individual host-seeking females in a laboratory wind-tunnel, we found that despite being a nocturnal species, *An. coluzzii* is highly responsive to a visually conspicuous object, but only in the presence of host-odour. Female mosquitoes approached and abruptly veered away from a dark object, which suggests attraction to visual cues plays a role in bringing mosquitoes to the source of host odour. It is worth noting that the majority of our recorded flight tracks consisted of highly stereotyped ‘dipping’ sequences near the ground, which have been mentioned in the literature, but never before quantified.

**Conclusions:**

Our quantitative analysis of female mosquito flight patterns within ~1.5 m of a host has revealed highly relevant information about responsiveness to visual objects and flight height that could revolutionise the efficacy of sampling traps; the capturing device of a trap should be visually conspicuous and positioned near the ground where the density of host-seeking mosquitoes would be greatest. These characteristics are not universally present in current traps for malarial mosquitoes. The characterisation of a new type of flight pattern that is prevalent in mosquitoes suggests that there is still much that is not fully understood about mosquito flight behaviour.

**Electronic supplementary material:**

The online version of this article (doi:10.1186/s13071-016-1609-z) contains supplementary material, which is available to authorized users.

## Background

Knowledge of sensory-controlled behaviour is of critical importance for designing tools to improve surveillance and control of insects responsible for the transmission of vector-borne diseases, as exemplified by the highly successful traps and lethal targets that control tsetse vectors of trypanosomiasis across Africa [1]. These devices utilise a range of long- and short-range sensory cues that bring flies to a bloodmeal host. There is a critical lack of such tools for sampling and controlling the world’s most important malaria vectors in the *Anopheles gambiae* complex [2, 3], responsible for 90 % of fatal malaria cases.

In recent years, 3D video-recording of mosquito behaviour under semi-natural conditions in a wind-tunnel has increased the precision with which flight manoeuvres can be characterised, thereby revealing details of species-specific mechanisms of host-location [4–7]. We applied this methodology to investigate two relatively neglected aspects of host-seeking in a nocturnal malarial species, *An. coluzzii* (a member of the *An. gambiae* species complex): (i) What is the pattern of flight tracks and flight height of female mosquitoes within ~1.5 m of a source of natural host odour? and (ii) Do mosquitoes use visual host cues to locate the source of host odours? These behaviours were chosen for their relevance to behaviour that might be exploited to enhance the efficiency and efficacy of lure and capture/kill devices. Thus far, behaviour-based traps for *An. gambiae sensu lato* typically catch less than a Human Landing Catch [8–11], in spite of using long-range olfactory stimuli to bring mosquitoes to the vicinity of the trap. It is possible that the efficiency of these traps could be increased by optimising the use of short-range host-associated stimuli based on more quantified knowledge of flight patterns near the host.

Whilst studying the endpoints of behaviour has led to the identification of attractive and repellent stimuli, crucial information about the process of host location can be obtained by observing the sequence of events that lead to this endpoint. For example, Cooperband & Cardé [12, 13] have shown through semi-field 3D video studies that the efficiency with which traps catch approaching mosquitoes depends on the positon of the odour release point in relation to the capturing (e.g. suction) device. This direct observation approach demonstrates the value of investigating mosquito flight behaviour in the immediate vicinity of trapping devices.

Adopting a similar approach, in our first experiment we investigated mosquito flight behaviour in the vicinity of a human host to gain insights into the sequence of behaviours that lead malaria mosquitoes to a specific host, with the aim of identifying characteristics of mosquito flight in the presence of a host that could be exploited to optimise the placement of a trap’s capturing device. We compared mosquito behaviour in two stages of host location: (i) during ‘ranging’ flight in clean air, before exposure to host cues; and (ii) during ‘host location’ flight to identify specific behaviours that are expressed only within ~1.5 m of a live host.

Previous studies of the effect of host odours on the flight behaviour of host-seeking mosquitoes have presented host odours as a discreet plume of odour, superimposed within a near-laminar air flow to minimise turbulence at the interface between host odours and clean air. This paradigm is useful for investigating odour-mediated opto-motor guided positive anemo-taxis, the mechanism by which mosquitoes fly toward a host from a distance, where host odours are the only indication that a host is present. Our objective, however, was to characterise host-seeking behaviour when a mosquito is within short range (less than ~2 m) of a whole human, as it would encounter a host *in situ*. In this context, the odour plume is better described as a broad field of host-odour laden air, extending from the ground upwards to a height that depends on the person’s size and posture (standing, sitting or prone). At this stage, the miasma of odour-laden air provides less reliable directional anemotactic cues; however, a wider range of host cues, such as visual and thermal stimuli, may be detected by the mosquito [7, 14, 15]. This range of host stimuli is of key relevance to the improved design of trapping methods.

Although mosquitoes are known to rely on visual cues to follow odour plumes, little is known about their response to visual host stimuli. Kennedy [16] was the first to show that a day-flying species, *Aedes aegypti*, compensates for wind-drift by opto-motor responses to features in their visual flow-field (visually guided anemotaxis), which has since been shown in the nocturnal species, *An. gambiae* (*sensu stricto*), at light intensities as low as starlight [17]. This behaviour demonstrates that the extraordinarily efficient light-gathering power of *An. gambiae* (*sensu lato*) eyes can compensate for their inherently poor visual acuity and resolution [18]. Less is known, however, about how the visual appearance of a host affects the flight behaviour of host-seeking nocturnal mosquitoes. The aim of the second experiment was, therefore, to identify the response of host-seeking females to a visually conspicuous object when in close proximity (~0.5 m) to a human host to determine whether visual cues might enhance the strength of attraction to lure and capture/kill traps.

## Methods

### Mosquitoes

A colony of *An. coluzzii,* previously M-molecular form of *An. gambiae* (*s.s.*) [19], was established with eggs from the colony at the Institut de Recherche en Sciences de la Santé/Centre Muraz in Bobo Dioulasso, Burkina Faso, and maintained at 26 ± 2 °C and 70 ± 5 % RH on a 12 h:12 h light:dark photocycle. Larvae were reared on Tetramin fish flakes (Tetra United Pet Group, Blacksburg, USA), adults were provided with 10 % sugar solution *ad libitum* and blood-fed on a human arm. Adult males and females were kept together in 30 cm sided cages.

### Flight arena

**Fig. 1:**
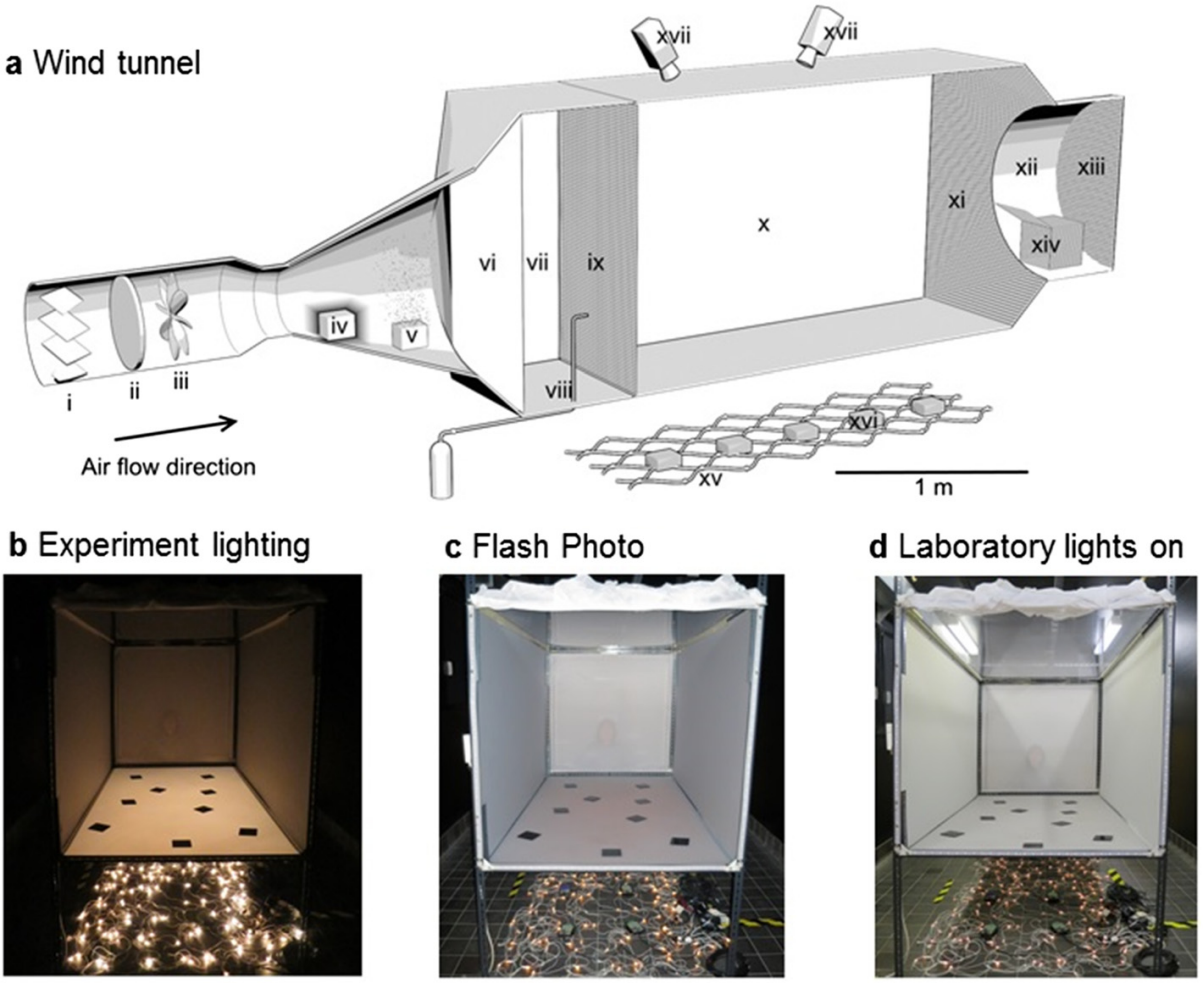
Wind tunnel and position of human host. **a** Three dimensional schematic of wind tunnel set-up, with a flight arena 1.2 × 1.2 × 2 m long. (i) shutter, (ii) charcoal filter, (iii) impelling fan, (iv) fan heater, (v) atomising humidifier, (vi) brushed-cotton cloth screen, (vii) odour delivery chamber, (viii) carbon dioxide source, (ix) upwind net screen, (x) flight arena, (xi) downwind net screen, (xii) insect release chamber, (xiii) terminal downwind net, (xiv) insect release cage, (xv) visible light-emitting diode (LED) array of white fairy lights on laboratory floor (~60 cm below flight arena), (xvi) black boxes containing infrared (IR) LEDs on laboratory floor and (xvii) cameras. Effects of lighting on appearance of arena floor and human host behind netting: **b** View of flight arena from downwind end with mosquito release unit (a-xii) removed, showing human volunteer just visible behind upwind screen, with experiment lighting only (IR-LEDs not visible and white LEDs brightly lit) and IR-pass filters on arena floor. **c** Same view taken with flash photography. **d** Same view with addition of laboratory room lights, showing position of IR-LED black boxes on laboratory floor and laboratory fluorescent ceiling lights visible through clear flight arena ceiling. Human host is sitting on stool in compartment a-vii with her waist ~5 cm below floor level of flight arena, mouth and extra CO_2_ at ~35 cm above floor level, behind white mosquito netting that obscures visual cues of the host

Our novel approach to the observation of flight behaviour within ~1.5 m of a human host under semi-natural conditions was to conduct our experiments in a flight arena that was tall and wide enough (1.2 m tall × wide × 2 m long; Fig. 1) to permit the use of a live human host, thereby creating a reasonably natural presentation of host odours and reducing the potential effects of the flight arena walls observed in some studies [7]. Under natural conditions the size and shape of the field of host-odour laden air would depend mainly on the size and orientation of the host and usually extend upward from ground level, as opposed to the more traditional laboratory-based host-odour plume presentations that typically consist of narrow plumes of odour, tens of centimetres in diameter and centred ~30 cm above ground level [4–7], more typical of broken-up packets of host odour that might be encountered > 1 m downwind of the host [20]. Our wind tunnel was designed to be large enough to provide corridors of clean air between the edges of the host-odour laden air and the walls and ceiling of the flight arena (see below).

**Fig. 2:**
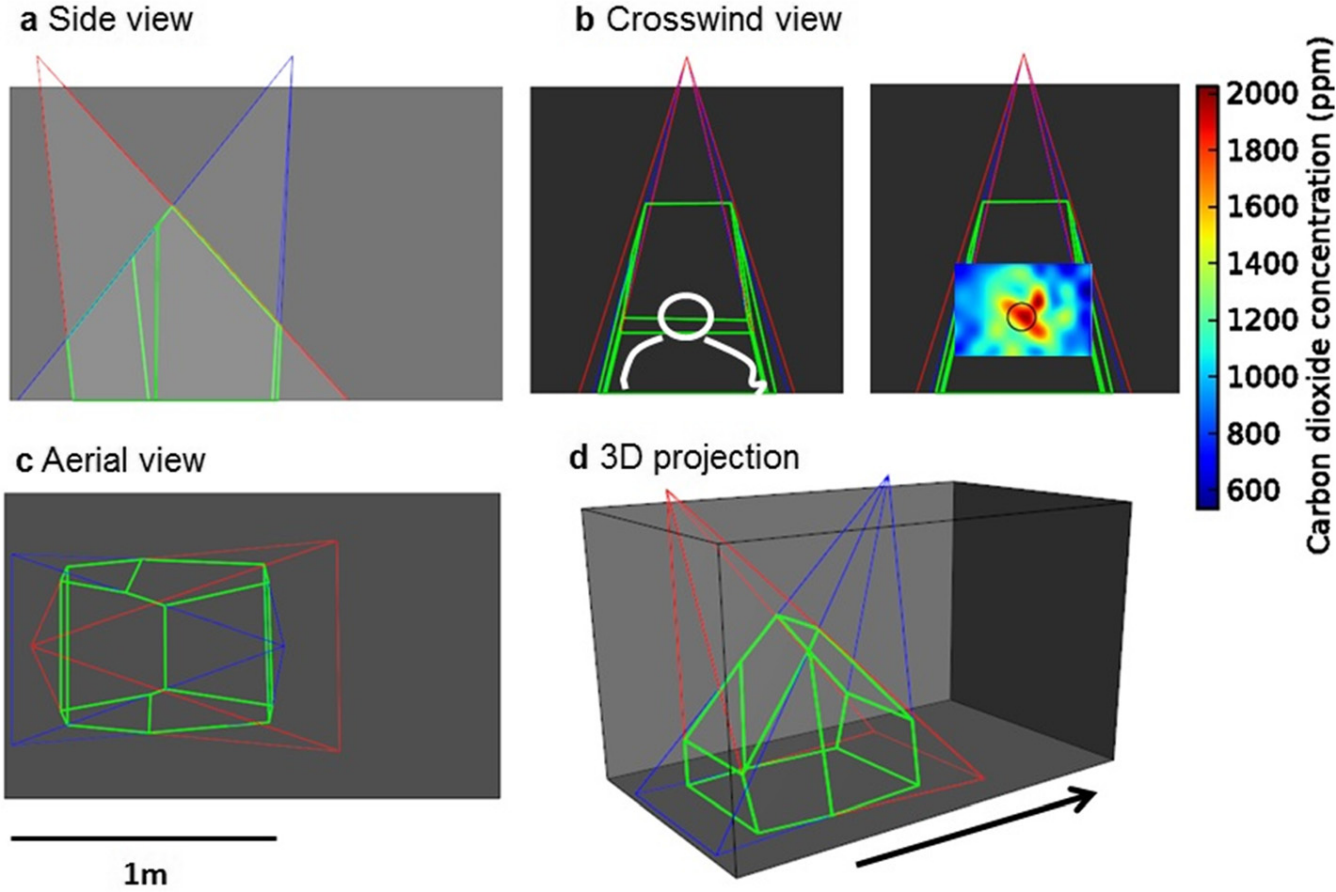
Schematic diagram of camera fields of view for recording mosquito flight within flight arena. Red and blue lines show camera fields of view, green lines show 3D capture area, grey shading represents flight arena (as seen in Fig. 1a-x). **a** Side view: 3D capture area ~85 cm at base and maximum height of 80 cm, upwind edge ~20 cm from upwind screen. **b** Crosswind view: Left panel shows human volunteer’s position (white line) and right panel shows CO_2_ concentrations. Human volunteer sat in upwind odour delivery chamber (Fig. 1a-vii), corresponding to the left side of panel **a**, **c** and **d**, with head positioned as shown and waist at flight arena floor level. Host’s breath and additional CO_2_ released at a height of ~35 cm. False colours show CO_2_ concentrations across wind ~50 cm downwind of host (i.e. centre of 3D field of view) and highest concentrations (1,400–2,000 ppm) at a height of ~30 cm. Hence, human odour effectively permeated middle ~60 cm of the flight arena up to a height of ~30 cm, corresponding to the area in which mosquitoes were observed. Clean air corridors of ~30 cm wide lined both side walls and the area above ~50 cm throughout the flight arena. **c** Aerial view. **d** 3D projection; arrow indicates direction of air flow

The side-walls and floor were white opal Perspex®, the roof was clear Perspex®, and the upwind and downwind ends were white netting (Fig. 1). The wind tunnel was kept at 25 ± 2 °C and 65 ± 5 % RH, with an air flow of 0.1 m s^-1^. ‘Clean’ air was drawn in by a fan from outside the building (inlet ~8 m from outdoor ground level), passed through a charcoal filter, heated, humidified, pushed through a screen of brushed cotton (Fig. 1a) and pulled through the flight arena by an extractor fan in the laboratory room at the downwind end, to create a near-laminar air flow for clean air experiments, and a relatively steady flow of air for host odour experiments, as visualised by the coherence of the CO_2_ plume ~50 cm downwind of the host (see text below and Fig. 2b).

Two high resolution analogue cameras (Fig. 1a-xvii: 1/3” CCD sensor, with infrared corrected, vari-focal auto-iris lens, *f*:1.0; SHC-735p; Samsung Electronics, Chertsey, UK) were mounted 1.3 m above the arena floor, with a 3D field of view indicated by the area within green lines in Fig. 2 (~60 cm wide × ~85 cm long at ground level). 3D flight coordinates of only one mosquito at a time were recorded, and only when no other mosquito was within the field of view (recording rate 50 Hz by TrackIt3D software [21], SciTrackS GmbH, Pfaffhausen, Switzerland) and video images were digitally recorded for later playback and data validation.

Back-lighting produced by ten infra-red (IR) lamps, each containing 90 IR light-emitting diodes (LEDs)s, placed 60 cm below the translucent floor of the flight arena (black boxes Fig. 1a-xvi, most visible on laboratory floor in Fig. 1d) enabled the video cameras to detect mosquitoes in silhouette [22, 23]. The wavelength spectrum of the IR-LEDs matched the wavelength sensitivity of the cameras (peak 840 nm, Tracksys, Nottingham, UK), but was beyond the wavelength sensitivity of mosquito eyes [17]. White light was provided by an array of 208 white LEDs (420–680 nm, fairy lights, Kontsmide, Sweden) placed on the laboratory floor (Fig. 1a-xv,b) alongside the IR lamps. The array of white LEDs produced a relatively evenly lit area of white light across the flight arena floor (2.0 × 1.5 m, Fig. 1b), similar in intensity to natural starlight reflected off the ground, as would naturally be experienced by nocturnal mosquitoes flying over sandy soil (~1.16 W m^-2^ [24]; flat-spectral response light meter [25]). Effectively, the white LEDs illuminated the mosquito’s field of view, illuminating visual cues used by flying insects to orient to wind direction [16, 17, 26], and the IR-LEDs illuminated the field of view of the cameras for video-photography.

Mosquitoes have the greatest light sensitivity and visual acuity in the anterio-ventral region of the eye, i.e. the region that would be looking at the ground slightly ahead of the mosquito during level flight [18, 27]. Accordingly, to enhance their detection of visual opto-motor cues, IR-pass plastic filters (10 cm sides × depth 0.3 cm, Instrument Plastics Ltd, UK) were placed randomly on the floor of the flight arena (Fig. 1b); wavelengths visible to mosquitoes do not pass through the disks, so they appear as dark areas to mosquitoes, but they are nearly invisible to the cameras, and, therefore, video-tracking was not impeded when mosquitoes flew across a filter (Additional file 1).

### Odours

Clean air passed through the wind tunnel for all experiments. For observations of behaviour with human odour present, a volunteer (GG) sat on a stool in the centre of the odour delivery chamber (Fig. 1a-vii) behind a front-lit screen of mosquito netting that obscured her visual features (Fig. 1a-ix). The volunteer sat on a stool in the middle of the chamber, with her mouth ~5 cm upwind of the screen and ~35 cm above the flight arena floor. Her waist was ~5 cm below the arena floor level, most of her torso was ~20 cm upwind of the screen and her legs were tucked under the stool that was below the odour delivery chamber and not visible in the flight arena. She was fully clothed, with only her forearms and head bare. To help maintain a reasonably steady flow of air, the volunteer was sealed into the chamber by a tight-fitting opal Perspex® panel around her torso at floor level of the flight arena. To increase the rate of activation [28], additional humidified CO_2_ roughly equivalent in concentration to a second person (4.8 % [29]) was provided at a rate of 5 l min^-1^ through silicone tubing and released directly adjacent to the volunteer’s mouth (Fig. 1a-viii).

Thus, host odours were released in a semi-natural context, over an area the size and shape of the volunteer’s outline in Fig. 2b, from the floor of the flight arena to the top of the volunteer’s head (~60 cm wide at floor level to ~20 cm wide the volunteer’s head), with a relatively coherent plume of CO_2_ plus human breath (mean height of ~30 cm and ~30–50 cm in diameter in the centre of the video-recorded area) superimposed within the larger area of human volatiles. Accordingly, when human odour was present, it effectively permeated the entire area that was video-recorded. There was also a clean air corridor (~30 cm wide) along both side walls and above ~50 cm throughout the flight arena.

To reduce daily variation in host odour composition [30], the same volunteer was used throughout and abstained from consuming alcohol or strong tasting foods and from using perfumed soaps and cosmetics 24 h prior to experiments.

The obstacle of a human positioned in the middle of the lower half of the wind tunnel will have had a considerable impact on the flow of air through the flight arena. Air turbulence was minimised, however, by the use of a push-pull system of air delivery through the wind tunnel (described above), as verified by mapping CO_2_ concentrations cross-wind in the middle of the videoed area (Fig. 2b); ~50 cm downwind of the source there was a relatively well-defined concentration gradient of CO_2_, with the highest concentrations covering a cross-section similar in dimension to the area of the volunteer’s head, centred ~30 cm above the floor, and ~5 cm below the height of the human volunteer’s mouth and the artificial CO_2_ source, as might be expected due to the relative density of CO_2_ compared to air (Fig. 2b). The mean concentration of CO_2_ was 1,060 ± 30 ppm (range = 791–3,462 ppm) at the centre of the plume over a 1 min sampling period (EGM-4 Environmental Gas Monitor, PP Systems, Amesbury, MA, USA). The overall mean background concentration of CO_2_, with no human or artificial CO_2_ present, was 460 ± 5 ppm (range = 439–511 ppm).

### Visual stimuli

A visually conspicuous object was placed in the centre of the 3D field of view, ~55 cm downwind of the host odour source behind the screen, and within the broad field of host odour. The object consisted of an IR transmitting plastic tile (20 × 20 cm × 0.3 cm thickness) held upright on a transparent Perspex® stand ~15 cm above the arena floor and perpendicular to the wind direction (Additional file 1). The IR-sensitive cameras detected mosquitoes even as they flew behind the apparently black IR transmitting tile. The responses to a transparent, colourless Perspex® tile of equal size and shape were also assessed to control for physical/mechanical cues created by disturbances to the flow of air in the vicinity of tiles of this shape and size that could potentially be detected by insects. The clear tile was effectively invisible; minimal reflections or glare were present because the only sources of light were beneath the wind tunnel floor and diffused by the opal Perspex® floor.

### Experimental procedure

Experiments were conducted in the first 3 h of the scotophase, when our colony of *An. coluzzii* are most responsive to host odours [28]. Access to sugar solution was removed 3 h prior to experiments to enhance responsiveness to host odours. For each trial a release cage containing five 5-10-day old female mosquitoes was placed at the downwind end of the flight arena (Fig. 1a-xiv) at the same height (~35 cm) as the centre of the human-breath/CO2 odour source (Fig. 2b) 5 min prior to the start of each trial. The release door was then opened without disturbing the mosquitoes to allow stochastic and odour-activated take-off. Flights were recorded for 10 min/trial, in either clean air or in the presence of the human volunteer. We assume that human odours were the dominant host stimuli detected by mosquitoes; visual host cues were obscured by the white screen and heat from the host is likely to have been dissipated to some extent by the screen and the distance to the closest point within the 3D video-area (> 20 cm from the host’s body). The flight arena was cleaned between assays and experiments with 75 % ethanol to avoid contamination with host odour between assays.

### Data acquisition and analysis

#### Activation

A test mosquito was considered to have been activated if it was not found in the release cage at the end of the trial period. The numbers activated of those released are reported for each experiment (Tables 1 and 2).

**Table 1:** Summary data from first experiment: effects of host odour on flight track parameters. Five females released per trial. Different letters denote significant differences between mean heights (Two-way ANOVA, *P* < 0.001)

Odour treatment	Number of trials	Mosquitoes activated/released	Number of tracks analysed	Track type (*n*)	Mean track duration ± s.e. (s)	Mean track height ± s.e. (cm)
Clean air	21	56/105	69	Smooth (20)	1.2 ± 0.08	11.8 ± 0.22^c^
				Tortuous (8)	3.6 ± 0.50	7.5 ± 0.20^b^
				Dipping (41)	1.3 ± 0.12	3.2 ± 0.15^a^
Host odour	18	63/90	49	Smooth (7)	1.9 ± 0.19	13.0 ± 0.29^d^
				Tortuous (17)	3.9 ± 1.13	13.6 ± 0.13^d^
				Dipping (25)	1.9 ± 0.22	3.4 ± 0.16^a^

**Table 2:** Summary data from second experiment: effects of host odour and visual stimulus on flight behaviour. Five females released per trial. Different letters denote significant differences in percentage of tracks showing a response to tiles (Fisher’s exact test; *P* < 0.05)

Tile type	Odour treatment	Number of trials	Mosquitoes activated/ released	Number of tracks analysed	Mean track duration ± s.e. (s)	Percent responded to tile
Clear	Clean air	20	47/100	44	1.9 ± 0.1	6.8^a,d^
	Host odour	21	88/105	54	1.9 ± 0.1	24.1^b^
Black	Clean air	21	66/105	51	1.8 ± 0.1	9.8^a^
	Host odour	14	56/70	53	1.9 ± 0.1	43.4^c^

#### 3D tracking

The following criteria were used to select tracks for analysis: > 0.5 s long (i.e. at least 25 data points), no segments of more than ten consecutive missing and/or errant data points, and < 30 % erroneous data points of either type. Errant or missing data points in useable tracks were interpolated using a cubic spline algorithm and track parameters were calculated in a custom built Python program (Python Software Foundation, Python Language Reference, version 2.6, USA).

#### Statistical analysis

In the first experiment, differences in flight parameters between mosquito tracks in clean air and with host odour present and between track types were compared with a two-way analysis of variance and *post-hoc* Tukey significance testing. Pearson’s chi-squared test with Yates’ continuity correction compared activation in clean air *versus* in host odour, and proportions of tracks of each flight type. In the second experiment, differences in the number of tracks demonstrating a response to the test tile were compared with Fisher’s exact test and flight parameters near the tile were compared in clean air *versus* in host odour using the *t*-test for unequal variances. The Chi-squared goodness of fit test compared distances at which mosquitoes turned away from tiles.

For the purposes of data analysis, each track is treated as an individual data point. Specific flight tracks cannot be attributed to individual mosquitoes, but at least one female per trial produced a flight track. Therefore, we have used the number of trials per treatment, rather than the number of flight tracks per treatment, as a more conservative value for ‘*N*’ when calculating degrees of freedom in the statistical analyses. Flight parameters were checked and found positive for normality both visually, via inspection of Q-Q plots and residual *versus* fitted values, and statistically, with the Shapiro-Wilk test of normality. All statistical analyses were undertaken in R [31].

## Results and discussion

### Activation

Over both experiments, a significantly greater percentage of mosquitoes flew out of the release cage when host odour was present; 54.5 % of mosquitoes in clean air and 78.1 % in host odour were activated within the 10 min observation period (pooled data from both experiments; Pearson’s Chi-squared test with Yate’s continuity correction, *χ*^*2*^ = 34.1, *df* = 1, *P* < 0.001; Tables 1 and 2). Taken together with the findings of similar wind tunnel studies on host-seeking in medically important mosquito species [4, 5, 32], it is clear that CO_2_ and host odour can stimulate activation in host-seeking females above the levels of activation expected during circadian phases of spontaneous activity; even over a 10 min observation period, a notable proportion of mosquitoes did not take off even in the presence of the activating stimulus of CO_2_ (21.9 % in host odour compared to 45.5 % in clean air).

### Effects of host odour on flight behaviour and location

**Fig. 3:**
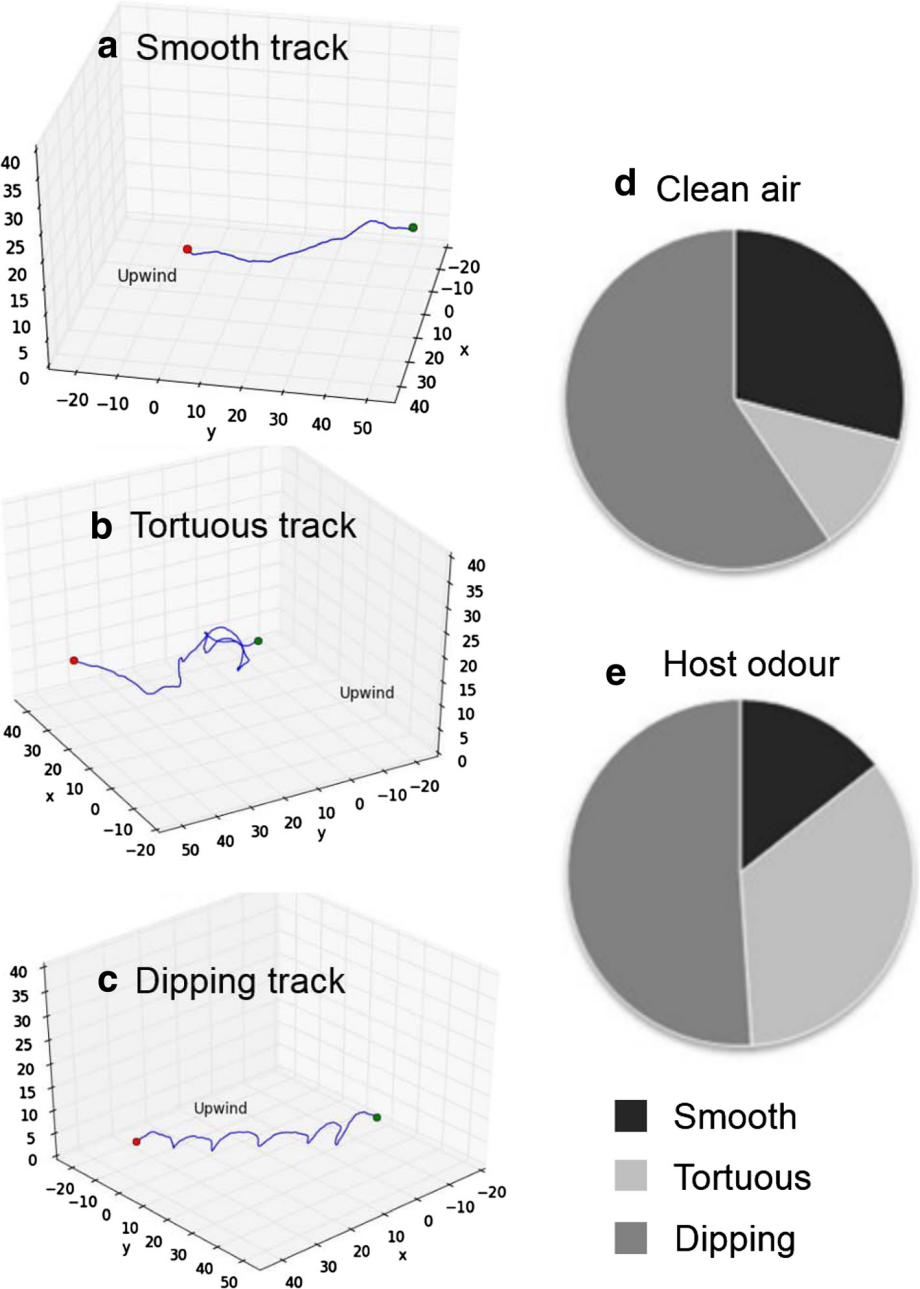
Examples of flight types: **a** Smooth, **b** Tortuous, and **c** Dipping flight tracks, and the relative proportions of each in **d** clean air and in **e** host odour. x, y and z axes are in cm. Numbers of tracks in clean air; smooth (20), tortuous (8) and dipping (41), and in host odour: smooth (7), tortuous (17) and dipping (25). Videos of each flight type can be found in Additional file 2 (Smooth flight in host odour), Additional file 3 (Tortuous flight in host odour) and Additional file 4 (Dipping flight in host odour)

In the first experiment, we observed three types of flight in both the presence and absence of a human host; smooth (Fig. 3a and Additional file 2), tortuous (Fig. 3b and Additional file 3) and a previously unquantified flight type we have defined as ‘dipping’ (Fig. 3c and Additional file 4). Consistent with studies on *An. gambiae s.s.* [32], *Aedes aegypti* [5, 14] and *Culex* spp. [6, 13], more tortuous flights were observed when the host was present than in clean air. Dipping flight consisted of highly stereotyped vertical oscillations (Fig. 3c) occurring close to the ground, rarely if ever touching the ground, and never flying higher than 3.5 cm. This behaviour has been noted in the literature [33], but not observed in previous wind tunnel studies, probably because it occurs close to the ground, and thus, out of range of most published 3D flight track recordings [4–6].

In addition, smooth and tortuous flights also occurred relatively close to the ground (< 13.6 cm), irrespective of host presence/absence. This is unlike previous studies, where these flight types were found much higher up, at the height of their respective artificial host odour plumes, typically 30 cm [4–7]. Although the height of the tracks was significantly higher in the presence of the host by 1.2 (smooth) and 6.1 cm (tortuous; Two-way ANOVA, *F*_(1,11784)_ = 599, *P* < 0.001, Table 1), their mean height was still considerably below the release cage height, which was at the level of the volunteer’s breath plus CO_2_ (~35 cm above the flight arena floor).

**Fig. 4:**
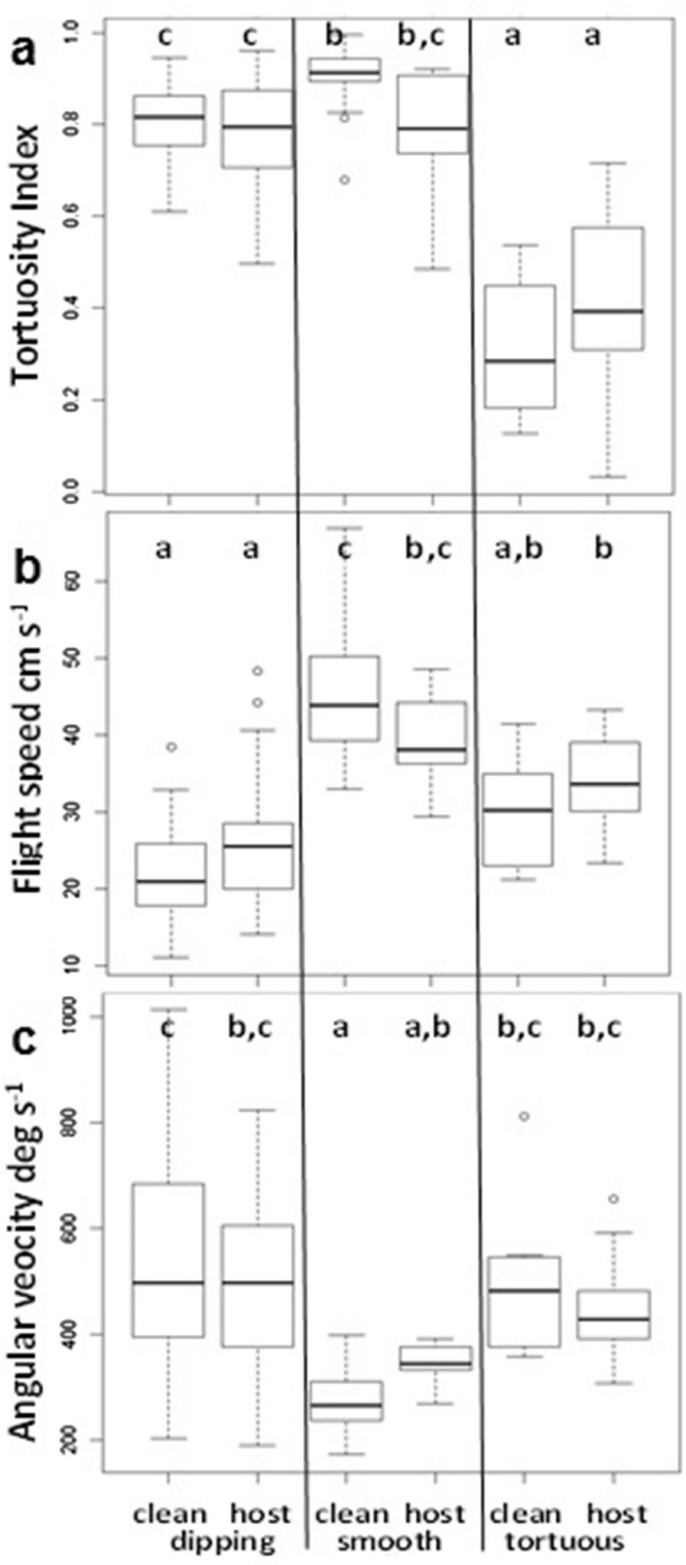
Comparison of flight types in clean air and in host odour. Solid lines represent median values, empty circles represent outliers. Bottom and top of the box show the 25^th^ and 75^th^ percentile, respectively. Whiskers show maximum and minimum values or 1.5 times interquartile range, whichever is the smaller. **a** Tortuosity index. **b** Flight speed. **c** Angular velocity. Different letters indicate boxes that are significantly different (Tukey *P* < 0.001, Two-way ANOVA). Numbers of tracks in clean air; dipping (41), smooth (20) and tortuous (8) and in host odour; dipping (25), smooth (7) and tortuous (17)

The flight parameters of smooth and tortuous flight were in accord with previous studies on mosquitoes [4–7]; smooth tracks were the straightest (tortuosity index = 0.9 ± 0.03), with the highest mean speed (45.1 ± 1.63 cm s^-1^) and the lowest mean angular velocity (268.5 ± 33.03° s^-1^; Fig. 4). Tortuous tracks were intermediate in speed (29.0 ± 2.57 cm s^-1^), with a relatively high angular velocity (497.8 ± 52.22° s^-1^) in all three planes, hence their extreme tortuosity (0.3 ± 0.04). Dipping tracks were unlike either of the others; they were relatively straight (tortuosity index = 0.8 ± 0.02), with the lowest mean speed of all flight types (22.5 ± 1.14 cm s^-1^), and although their mean angular velocity (531.9 ± 25.71° s^-1^) was similar to that of tortuous tracks (Fig. 4), turning was limited almost exclusively to the vertical plane (Fig. 3b, c).

The most notable effects of host presence were observed in smooth tracks, with significantly greater mean tortuosity and angular velocity, but slower mean flight speed (Fig. 4), and greater mean flight height of smooth and tortuous tracks (Table 1). Host presence had no significant effects on these flight track parameters in dipping flight.

The majority of tracks recorded were dipping flights irrespective of the presence/absence of the host (55.9 %, Table 1, Fig. 3d, e), indicating this flight pattern may play a role in the behaviour of both ranging and odour-plume following mosquitoes. The presence of the host had a significant effect on the relative proportions of flight types, trebling the overall percentage of tortuous tracks (11.6 to 34.7 %), and decreasing the percentage of smooth (29.0 to 14.3 %) and dipping tracks (59.4 to 51.0 %; Pearson’s *χ*^*2*^ = 10.28, *df* = 2, *P* = 0.006; Table 1, Fig. 3d, e).

Overall, dipping flights were less prevalent when mosquitoes were exposed to a host, and tortuous flights at higher altitudes were more common. Nonetheless, mosquitoes notably flew closer to the ground (mean flight track heights < 14 cm) than might have been expected from previous wind tunnel studies [4–7].

### Analysis of dipping flight

Although dipping-type flights have been described in the literature, this is the first quantitative analysis of their flight parameters. Most recently, Parker et al. [33] recorded similar patterns of behaviour over the surface of an occupied bednet under field conditions. The potential significance of dipping flight as a fundamental type of behaviour may have been overlooked and therefore it merits a more detailed analysis.

**Fig. 5:**
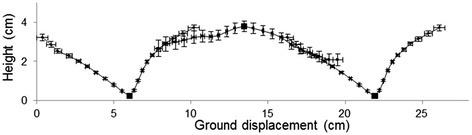
Reconstructed dipping flight in host odour. Cumulative horizontal displacement flown per 20 ms intervals against height (mean ± standard error, s.e.), based on data from *n* = 84 peaks and *n* = 82 troughs (taken from 25 tracks) and a calculated trough-trough period of ~0.5 s. Square indicates minima of trough or maxima of peak, to which each track segment was aligned. Small error bars leading into and out of trough show highly consistent ground speed and flight angle to ground of tracks, whereas there is greater variation between tracks in flight trajectories of peaks

The oscillations of dipping flight consisted of sharp troughs that nearly touched the ground (Fig. 5); in the presence of a host the height of trough minima was only 0.2 ± 0.06 cm (*n* = 82). These troughs were followed by broad peaks ~4.0 cm above the troughs. Accordingly, the mean amplitude of dips (trough to peak) was significantly greater when the host was present (2.8 ± 0.1 cm) than absent (1.9 ± 0.1 cm; ANOVA: *F*_(1,211)_ = 17.19, *P* < 0.001) and the mean duration of trough-to-trough cycles in the presence of the host was significantly longer (0.51 ± 0.02 s) than in clean air (0.43 ± 0.02 s; ANOVA: *F*_(1,211)_ = 5.27, *P* = 0.023). The effects of the host, whilst significant, are minimal in their impact on the overall regularity of the pattern of dipping flight.

Figure 5 shows a representation of mosquito displacement during dipping flight in the presence of the host. The highly consistent pattern of troughs was produced by mosquitoes maintaining a constant angle of descent and a relatively constant ground speed as they approached the minima of the trough, with a mean descent angle of 26.0 ± 2.03°. This was followed by a sharp upward turn of 70.5 ± 2.48° made at the trough minima, a steep ascent at 50.7 ± 2.71° for the first 0.1 s, and then a reduced angle of ascent of 26.4 ± 2.75° as mosquitoes came out of the trough. Peaks were broader than troughs overall, with shallower turns at the apex of peaks than at the bottom of troughs and more constant flight speeds across the peaks. The highly consistent pattern of dipping flight and its ubiquity across trials is indicative of a functional behaviour, the purpose of which is unclear.

### Effects of a host on mosquito response to a visually conspicuous object

In the second experiment, female mosquito flight tracks were recorded in the presence of an upright black tile, in clean air or in the presence of a host, to determine whether a visually conspicuous object is attractive to mosquitoes stimulated by volatile odours emanating from a host. To isolate potential visual cues of the object from physical cues (such as distortion of air flow), two tiles were tested; a black tile of high visual contrast and a colourless, transparent tile, similar in all respects, but lacking in visual features. Hence, both tiles would have had a similar effect on air currents, but the black tile would be visually more conspicuous than the clear tile.

We found that mosquitoes were highly sensitive to the visual stimuli from the object in host odour; nearly half (43.4 %, Table 2) of flight tracks showed a response to the black tile (Additional file 1; video of flight around a tile). Mosquito 3D flight tracks demonstrated a characteristic directed response to the black tile, but only in the presence of a host; they flew directly toward the face of the tile, coming within a few centimetres of its surface and then rapidly flew up and away without contacting it. These tracks were characterised as “Responders” and according to the following salient flight parameters: the mosquito flew toward the tile and came within 0 to 15 cm of the tile’s surface and executed a change in angular velocity of > 90° at its minimum distance from the tile (i.e. when the mosquito was closest to the tile). Remarkably, around a quarter (24.1 %, Table 2) of tracks also responded to the physical presence of the clear tile in the presence of a host, although significantly fewer responded to the clear tile than to the black tile (Fisher’s exact test, *P* < 0.05), and the flight parameters differed (described below). However, in the absence of a host, less than 10 % of mosquitoes responded to either tile (9.8 % black tile, 6.8 % clear tile; not significantly different, *P* = 0.721, Table 2). This indicates in *An. coluzzii* an odour-dependent responsiveness to visual stimuli; without host odour they did not respond to visual cues.

**Fig. 6:**
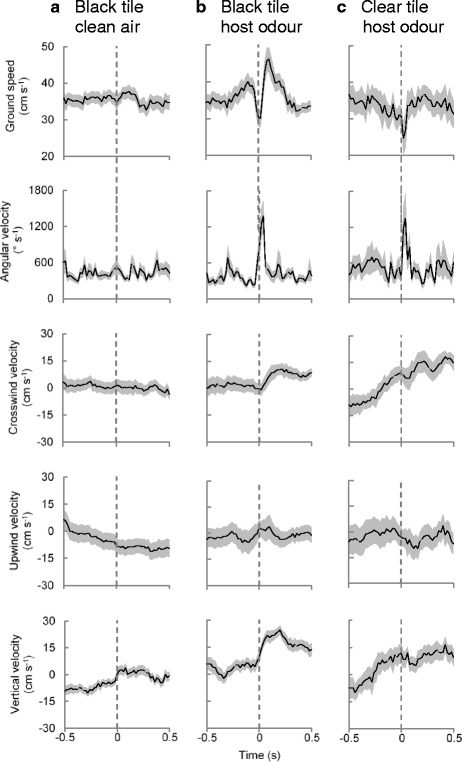
Flight track parameters of female mosquitoes near objects. **a** Non-responders to high contrast (black) object in clean air (i.e. baseline values, *n* = 46). **b** Responders to black object in host odour (*n* = 23). **c** Responders to clear object in host odour (*n* = 13). Plots show mean values (*solid line*) and standard errors (*grey envelope*), and are aligned to ‘0 s’, the moment a track came closest to the object (*dashed line*)

A quantitative analysis of these tracks reveals some interesting features of flight control. Tracks showing a response to the black tile in host odour (Fig. 6b) had rapidly decreasing mean ground speeds as they approached the tile, decelerating from 40 cm s^-1^ to 30 cm s^-1^ in the 0.1 s prior to arriving at their closest position to the black tile. Mosquitoes then accelerated at ~80 cm s^-2^, reaching a mean speed of 46 cm s^-1^ within 0.1 s of moving away from the tile, whilst also increasing mean angular velocity at a significantly greater rate compared to clean air tracks over the same timeframe (Fig. 6a, b, Table 3A at 0.04 s, i.e. at maximal response). The slowest speeds coincided with the closest point to the tile, after which speed increased for ~0.08 s as the mosquitoes moved away from the tile. Angular velocity increased more than five-fold, with a mean angular acceleration of 12,500° s^-2^ between 0.04 s before and 0.04 s after the closest point to the tile, before returning rapidly (within 0.02 s) to a level comparable with their approach to the tile (Fig. 6b). The mean velocity in vertical displacement was significantly greater in host odour than in clean air when a black tile was present (Table 3A). At 0.5 s of reaching their closest point to the black tile, mosquitoes in host odour treatments had returned to similar speeds and angular velocities as found at 0.5 s before this point, however, both their vertical and crosswind velocity remained significantly higher (Table 3A, Fig. 6b), representing a continued movement away from the black tile. In contrast, mosquitoes that flew near the black tile in clean air but did not respond to it were characterised as having relatively constant mean ground speeds and angular velocities (normalized to their minimum distance from the tile; Fig. 6a).

**Table 3:** Statistical comparisons of the effects of odour and appearance of an object on mean flight parameters. (A) Female mosquitoes with black tile present, in clean air *vs* host odour (Fig. 6a, b). (B) Female mosquitoes in host odour with black *vs* clear tile present (Fig. 6b, c). 3D positional data were aligned to the time at which a track came nearest the tile (0.0 s) and statistical comparisons correspond to the maximal reading for the particular metric tested (at -0.5, 0.1 and 0.5 s for speed and velocities, and at -0.5, 0.04 and 0.5 s for angular velocity, significant results in bold). All values were calculated using the *t*-test for unequal variance

(A) Black objects in	-0.5 s			0.1 s			0.5 s		
clean *vs* host odour	*df*	*t*	*P*	*df*	*t*	*P*	*df*	*t*	*P*
Speed	42.3	-0.56	0.57	37.5	2.29	**< 0.05**	54.5	-0.52	0.59
Cross-wind velocity	42.5	-0.52	0.60	57.9	1.25	0.21	57.2	3.23	**< 0.01**
Upwind velocity	43.0	-1.40	0.16	45.1	1.57	0.12	57.9	1.30	0.19
Vertical velocity	42.0	3.49	**< 0.01**	50.8	4.87	**< 0.001**	48.7	3.44	**< 0.01**
	-0.5 s			0.04 s			0.5 s		
Angular velocity	32.1	-0.74	0.46	29.1	3.58	**< 0.01**	48.4	0.53	0.59
(B) Black *vs* clear	-0.5 s			0.1 s			0.5 s		
objects in host odour	*df*	*t*	*P*	*df*	*t*	*P*	*df*	*t*	*P*
Speed	15.0	-0.54	0.59	29.9	2.26	**< 0.05**	13.2	0.53	0.59
Cross-wind velocity	20.6	1.15	0.26	20.8	-0.38	0.70	17.8	-1.22	0.23
Upwind velocity	17.1	0.21	0.83	29.8	1.19	0.24	22.3	0.78	0.44
Vertical velocity	13.2	1.77	0.09	26.6	2.70	**< 0.05**	24.1	0.78	0.44
	-0.5 s			0.04 s			0.5 s		
Angular velocity	19.8	0.17	0.85	19.6	0.06	0.94	15.61	-1.16	0.26

Tracks of mosquitoes that responded to the black or clear tile in host odour (Fig. 6b, c) reached similar peak mean angular velocities during their turn away from the tile within 0.04 s after reaching their closest point to it (Table 3B, *t*-test for unequal variance, *t* = 0.06, *P* > 0.9). Tracks showing a response to the black tile began to turn earlier, however, by increasing their angular velocity ~0.01 s before reaching their closest point to the tile, whereas those responding to the clear tile increased their angular velocity only once they had reached their closest point to it (Fig. 6c). The most striking difference in response to the two types of tile was observed in flight speeds (Fig. 6b, c, Table 3B); mosquitoes responding to the clear tile did not show a surge in ground speed as they came closest to the tile or decrease in their speed after leaving the tile. Also, the majority of subsequent displacement away from the clear tile occurred in a crosswind direction, with a significantly less steep vertical displacement than seen in response to the black tile (Table 3B).

**Fig. 7:**
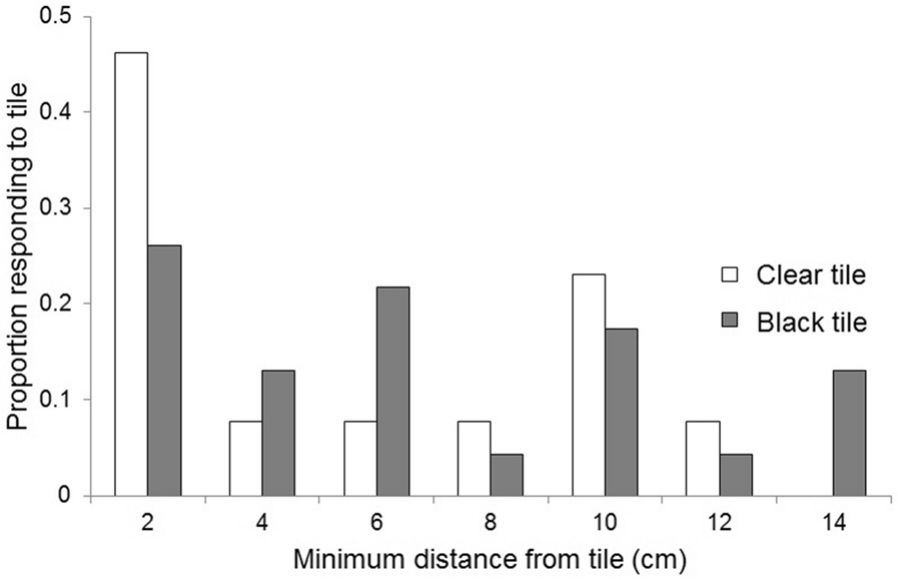
Distribution of minimum distances at which mosquitoes turned away from object in host odour. The distribution of responses to the clear object is significantly different from an equal distribution (Chi-squared goodness of fit test, *n* = 13, *df* = 6, *χ* ^*2*^ = 13.38, *P* < 0.05), whereas the distribution of responses to the black object is not (*n* = 23, *df* = 6, *χ* ^*2*^ = 6.52, *P* = 0.36)

In host odour there was no significant difference in the mean distance at which responding mosquitoes turned away from the clear (4.5 ± 1.13 cm) or the black tiles (5.4 ± 0.85 cm; ANOVA, *F*_(1,36)_ = 0.4, *P* = 0.5). However, a histogram of these distances shows that nearly half of the individuals flying towards the clear tile executed a turn away from it only within 2 cm of its surface (Fig. 7), a response spread that is significantly different to an equal distribution across the range (Chi-square goodness of fit test, *χ*^*2*^ = 13.38, *n* = 13, *df* = 6, *P* = 0.037), whereas insects flying towards the black tile show a non-significantly different response across the range of distances, up to 15 cm away (*χ*^*2*^ = 6.52, *n* = 23, *df* = 6, *P* = 0.36).

These results suggest that the visual expansion of the black tile was sufficient to instigate avoidance manoeuvres at a distance from it, as seen in visual responses of *Drosophila melanogaster* [34]. For the clear tile, the majority of mosquitoes turned away within the last few cm before contacting it, indicating that non-visual cues operating over very close range may provide the stimuli needed to avoid collision.

## Conclusions

The main outcome of our study was the discovery that host-associated olfactory stimuli modulate the response of mosquitoes to visual stimuli; *An. coluzzii* females make oriented flights toward a visually conspicuous object in the presence of human odours. There was no evidence, however, of attempts to land on the object, suggesting that visual and olfactory cues are not sufficient to trigger landing responses.

The findings reported here demonstrate that, in spite of their poor resolution, nocturnal mosquitoes are highly responsive to visual cues, even in light levels equivalent to starlight, when concurrently stimulated by host odours. Female *An. coluzzii* mosquitoes in an odour plume flew rapidly toward an upright black tile, but turned sharply (accelerating at 12,500° s^-2^) within 15 cm of colliding with it, and accelerated rapidly (80 cm s^-2^) up and away from it in a highly consistent flight pattern. This is consistent with findings for the diurnal/crepuscular mosquito *Ae. aegypti* [14], which showed a response to black areas on the floor of a wind tunnel, especially when host odour was present. This has profound significance for the design of future sampling and control devices for nocturnal malaria vectors; a greater proportion of mosquitoes that have been lured to the general vicinity of a trap by natural or synthetic odour baits may be induced to fly in close proximity to the collection mechanism if a trap incorporates behaviourally relevant visual stimuli, thus increasing the likelihood that an individual will be caught.

We also discovered that > 50 % of observed flight tracks in Experiment 1 were dipping flights. By direct observation, dipping appeared to be similar to the ‘dancing’ flight of ovipositing mosquitoes [35, 36] and the ‘bouncing’ flight of host-seeking *An. gambiae* (*s.s.*) over bednets [33]. Dipping flight may have a similar role in all three contexts, e.g. a mechanism first proposed by Gillette [37] for non-visual assessment of air current direction.

That prevalent behaviours such as those described here have gone unreported highlights that our knowledge of mosquito flight behaviour is in large part incomplete. The discovery that mosquitoes are highly responsive to host visual-cues primarily when stimulated by host olfactory-cues and that they fly close to the ground (< 14 cm) even in the presence of host odours could provide highly advantageous components to traps designed to attract host-seeking malarial mosquitoes. Field validation of these behavioural findings should therefore be a priority. Future studies should consider the integrated behavioural effects of multiple host-associated stimuli, which could yield results with direct relevance to the design of bio-rational monitoring and control tools.

## Additional files

Additional file 1: “Flight around black tile in host odour”: video file showing an aerial view of the wind tunnel flight arena in both normal daylight and infrared lighting, followed by a typical mosquito flight track responding to a black tile in host odour and a 3D reconstruction of this flight track. (WMV 7788 kb) Additional file 2: “Smooth track in host odour”: video file showing a 3D reconstruction of a typical smooth mosquito flight track in host odour. (AVI 4645 kb) Additional file 3: “Tortuous track in host odour”: video file showing a 3D reconstruction of a typical tortuous mosquito flight track in host odour. (AVI 5295 kb) Additional file 4: “Dipping track in host odour”: video file showing a 3D reconstruction of a typical dipping mosquito flight track in host odour. (AVI 5027 kb)
